# The effects of multimodal lifestyle interventions on blood pressure, body weight, and waist circumference in hypertensive patients: a network meta-analysis

**DOI:** 10.3389/fpubh.2026.1720695

**Published:** 2026-03-02

**Authors:** Ting Yao, Qiao Hu, Dongrui Miao, Zheyuan Xia, Miaomiao Li, Xiang Wang, Huiling Zhang

**Affiliations:** 1 School of Nursing, Anhui University of Chinese Medicine, Hefei, Anhui, China; 2 Department of Burns, The First Affiliated Hospital of Anhui Medical University, Anhui, China; 3 Key Laboratory of Geriatric Nursing and Heath, Anhui University of Chinese Medicine, Hefei, China

**Keywords:** hypertension, network meta-analysis, randomized controlled trials, waist circumference, weight loss

## Abstract

**Objective:**

To employ a network meta-analysis methodology to compare the effects of different lifestyle interventions on weight control and blood pressure in hypertensive patients, thereby providing evidence-based guidance for nursing practice.

**Methods:**

A systematic search was conducted across PubMed, Web of Science, the Cochrane Library, Embase, the Chinese Biomedical Literature Database (CBM), China National Knowledge Infrastructure (CNKI), Wanfang Data, and VIP Database, up to February 2025, concerning the effects of different lifestyle interventions on body weight in hypertensive patients. Study selection, data extraction, and methodological quality assessment adhered to evidence-based research standards using a network meta-analysis approach within a frequency framework. Statistical analysis was performed using Stata 16.0 software.

**Results:**

Twenty studies involving 5,248 hypertensive patients were included, with interventions covering four fundamental lifestyle modifications and their combined protocols. The overall quality of the included literature was high. Results from the network meta-analysis indicated that, compared with routine care, single or combined interventions based on telemedicine support, dietary modification, exercise, or peer support all helped obese hypertensive patients achieve weight loss and improve blood pressure control. After comprehensively evaluating the overall improvement in patients’ weight and blood pressure status, combined interventions integrating diet, exercise, and peer support may represent one of the more suitable management strategies.

**Conclusion:**

Management programmes incorporating telemedicine, dietary intervention, exercise intervention, or peer support all contribute to achieving weight loss and blood pressure reduction goals in obese hypertensive patients. Among these, combined interventions integrating diet, exercise, and peer support may represent a favorable option.

**Systematic review registration:**

https://www.crd.york.ac.uk/PROSPERO/view/CRD42024575811, identifier CRD42024575811.

## 1 Introduction

Hypertension and obesity are two chronic diseases with continuously rising global prevalence rates (1, 2), often coexisting to produce a significant synergistic pathogenic effect (3). Obesity substantially increases the risk of hypertension through multiple pathophysiological mechanisms, including inflammation, insulin resistance, and sympathetic nervous system activation (4), while weight reduction has been proven to yield clear blood pressure-lowering benefits. Research indicates that weight loss produces a substantial blood pressure-lowering effect, with each kilogram of weight reduction associated with an average decrease of approximately 1 mmHg in systolic blood pressure (5). Therefore, for patients with obesity and hypertension, effective weight management constitutes one of the core strategies for blood pressure control.

Lifestyle interventions have become the preferred approach for weight management due to their safety, cost-effectiveness and sustainability. Current mainstream interventions encompass multiple modalities, including dietary changes, exercise, and telemedicine support, varying in specific composition, intensity, and implementation formats. Although clinical studies confirm that combined interventions integrating exercise and dietary adjustments can achieve dual benefits of weight loss and blood pressure reduction, the relative efficacy of different single or combined lifestyle intervention programmes, and which approach offers greater advantage in improving composite outcomes of weight and blood pressure, remains inconclusive.

While traditional meta-analyses permit comparative assessments between single interventions and control groups, they cannot directly compare or quantitatively rank multiple intervention strategies. To evaluate the effects of diverse lifestyle interventions on body weight and blood pressure in obese hypertensive patients, and to identify optimal strategies, network meta-analysis emerges as an essential research methodology. This approach integrates both direct and indirect evidence, enabling multiple comparisons and ranking. However, precisely because existing lifestyle intervention programmes are diverse and clinical selection requires multiple comparisons and prioritisation, traditional meta-analysis methods fail to meet this core requirement. Therefore, a comprehensive, systematic network meta-analysis remains lacking to quantitatively compare and rank the efficacy of different lifestyle interventions and their combination strategies for obese hypertensive patients. To address this clinical question, this study employs a network meta-analysis approach grounded in a frequency framework. It quantitatively compares and ranks the efficacy of diverse lifestyle intervention strategies in controlling weight and blood pressure, aiming to identify superior intervention protocols. This provides evidence-based guidance for personalized weight management in hypertensive patients with obesity.

## 2 Methods

### 2.1 Search strategy

Computer retrieval from PubMed, Web of Science, the Cochrane Library, EMbase, CBM, CNKI, Wanfang Data, and VIP Database, spanning from their inception to February 1, 2025, employing a combination of subject terms and free terms. The English search strategy, exemplified by the PubMed database, is structured as follows:(((((“Hypertension”[Mesh]) OR (Blood Pressure*, High[Title/Abstract])) OR (High Blood Pressure*[Title/Abstract])) AND ((((“Weight Loss”[Mesh]) OR (Loss*, Weight[Title/Abstract])) OR (Weight Reduction*[Title/Abstract])) OR (Reduction*, Weight[Title/Abstract]))) AND ((((((((((((“Diet”[Mesh]) OR (“Friends”[Mesh])) OR (Friendship*[Title/Abstract])) OR (Companion*[Title/Abstract])) OR (Acquaintance*[Title/Abstract])) OR (“Telemedicine”[Mesh])) OR (“Movement”[Mesh])) OR (“Life Style”[Mesh])) OR (Lifestyle*[Title/Abstract])) OR (Life Style Induced Illness[Title/Abstract])) OR (Lifestyle Factor*[Title/Abstract])) OR (Factor, Lifestyle[Title/Abstract]))) AND (((randomized control[Title/Abstract]) OR (randomized controlled trial[Title/Abstract])) OR (RCT[Title/Abstract])) AND (eng[la]).

### 2.2 Selection of studies

Two researchers utilized EndNote software for literature management. Following the initial reading and screening, a secondary screening was performed to extract relevant data from the included studies. The extracted data encompassed the first author, publication date, country or region, age range, target intervention population, sample size, duration of intervention, intervention methodologies, and outcome metrics. In cases of divergent opinions regarding the literature, consultations were conducted with a third party.

### 2.3 Population

The following criteria must be met concurrently: (i) According to the latest clinical practice guidelines issued by the American Heart Association regarding blood pressure classification, blood pressure exceeds normal values (systolic pressure ≥120 mmHg and diastolic pressure ≥80 mmHg) (6). (ii) Body Mass Index (BMI) > 25 kg/m^2^. (iii) Exclude gestational hypertension.

### 2.4 Interventions and comparisons

To compare the efficacy of different lifestyle interventions for weight management, this study categorized the identified interventions into four fundamental types: dietary control, exercise training, peer support, and telemedicine support. Within the included randomised controlled trials, intervention groups typically employed one or a combination of these fundamental categories (e.g., telemedicine support + dietary control, telemedicine support + peer support). For the network meta-analysis, each unique intervention combination was defined as an independent analytical node. For instance, “dietary control + exercise training” was treated as a distinct intervention measure, differentiated from single measures like “dietary control” or “exercise training” and from combinations such as “dietary control + peer support”. Ultimately, the network comprised 10 nodes, encompassing diverse forms ranging from monotherapies to multi-component combined interventions, enabling a comprehensive assessment of the relative efficacy of different intervention strategies.

### 2.5 Outcome indicators

The primary outcome measures were reductions in patients’ body weight and blood pressure. Secondary outcome measures included waist circumference reduction and blood pressure reduction effectiveness rate. The latter was defined, based on the characteristics of the included studies, as the ratio of individuals achieving target blood pressure control following intervention to the total number of individuals undergoing intervention.

### 2.6 Study exclusion criteria

Studies in which pharmacologic interventions or surgical treatments were used in combination in the intervention group were excluded. Duplicate publications; non-Chinese and English literature; no access to original, raw data.

### 2.7 Risk of bias assessment

Risk of bias evaluation was independently performed by 2 investigators using the Cochrane Handbook version 5.1.0 Risk of Bias Assessment Scale for Randomized Controlled Trials to evaluate the quality of the literature and to cross-check. The risk assessment tool included 7 items: randomized sequence generation, allocation concealment, blinding of subjects and personnel, blinding of outcome assessors, incomplete outcome data, selective reporting of results, and other risks of bias. Each item was assessed as “low risk,” “high risk” or “uncertain.” If all of the items were “low risk,” they were graded as A, some of them were graded as B, and none of them were graded as “low risk.” This tool does not employ weighted scoring; the final results are presented via a literature risk assessment diagram generated using Review Manager 5.4 software.

### 2.8 Data analysis

Prior to commencing the meta-analysis, this study extracted baseline characteristics (including age, blood pressure, and body weight) from each group within the included studies. Box plots depicting the distribution of baseline characteristics across multiple groups were generated and visually compared. This enabled an assessment of whether significant systematic differences existed in key baseline variables between different studies, serving as preliminary evidence to determine whether the transferability hypothesis held for the network meta-analysis. Should the distributions of baseline characteristics exhibit high overlap across studies, with similar medians and dispersion, this would indirectly support the comparability of populations across different research settings, thereby satisfying the prerequisite for indirect comparison. This methodology provides crucial foundational support for subsequent evidence synthesis based on the consistency model.

Continuous outcome measures in this study (body weight, blood pressure, and waist circumference) were expressed as mean differences from baseline and their standard deviations. For studies reporting mean values and standard deviations at baseline and follow-up, we directly calculated the mean difference and standard deviation. Where the SD of the change was not directly provided, it was estimated using the method recommended by the Cochrane Handbook (7): given the strong correlation between pre- and post-intervention values for measures such as weight, the correlation coefficient between baseline and follow-up values was assumed to be r = 0.8. If the standard deviation was not reported, it was inferred using standard statistical conversion methods [employing the approach of Hozo et al. (8)] based on the reported *p*-value, confidence interval, or data range. The dichotomous outcome measure in this study was the rate of effective blood pressure control, with data extracted as event counts (number of individuals achieving target blood pressure) relative to the total sample size. Effect sizes were expressed as odds ratios (OR) with 95% confidence intervals. An OR > 1 indicates a higher blood pressure control rate in the intervention group compared to the control group. Where the original study did not directly report the OR, we calculated the OR value based on the number of individuals achieving target blood pressure control and the total number of participants.

The network meta-analysis employed a random-effects model capable of handling multiple group comparisons with interrelatedness. This model ensured accurate standard error estimation by integrating the covariance arising from shared controls across intervention groups within the same study. To address heterogeneity between studies, a random-effects model based on the generalised DerSimonian-Laird estimator was adopted (9). Sensitivity analyses were conducted to assess the potential impact of model selection on results. In the primary analysis, we employed a random-effects model to fully account for inter-study heterogeneity. As a sensitivity analysis, we simultaneously refitted the data using a fixed-effects model and compared the effect size estimates and intervention ranking probabilities obtained from both models to test the robustness of the primary conclusions. Network inconsistency was assessed through the following steps: first, a global Wald test was performed using the Design-by-Treatment Interaction model (significance level set at *p* < 0.05); if inconsistency was indicated, local tests were further conducted using node splitting. Results from the consistency model were presented for the primary analysis. For included studies featuring multiple intervention groups within the same predefined category, we combined their means, standard deviations, and sample sizes prior to analysis to form a single treatment group, following the recommended method in the Cochrane Handbook (7). The ranking of relative treatment effects was achieved using the Sum of Areas under Cumulative Ranking Probability (SUCRA) values, where higher SUCRA values indicate superior effectiveness. Publication bias was assessed by visually comparing the symmetry of the correction funnel plot.

## 3 Results

### 3.1 Studies search results

**Figure 1:**
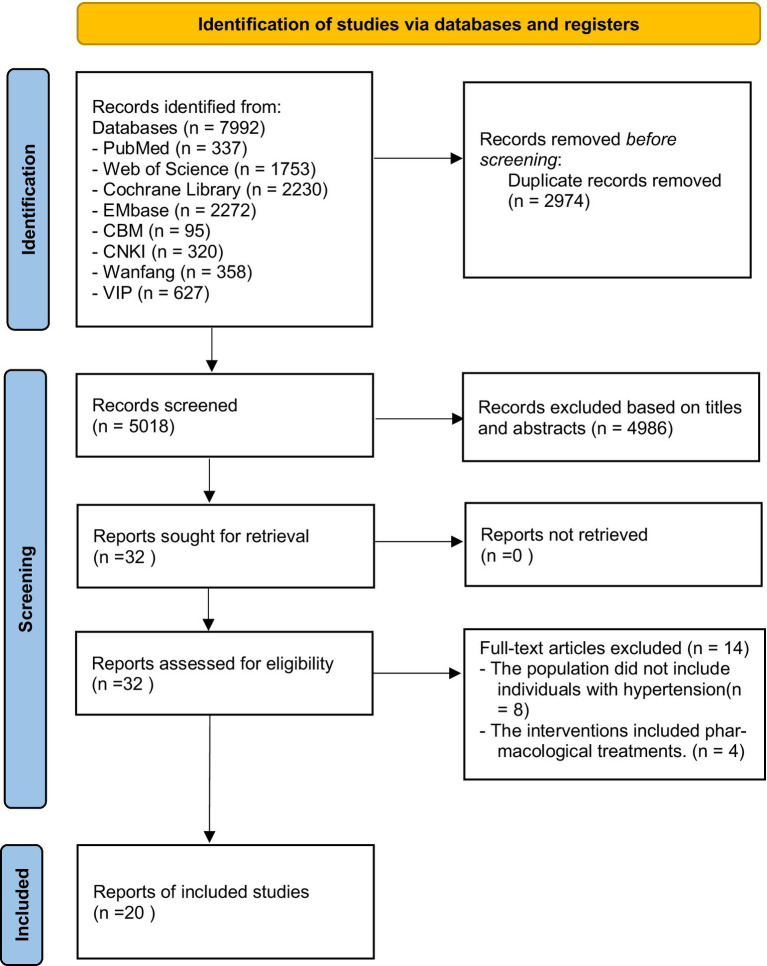
The flow chart of literature screening.

The initial search yielded 7,992 articles. After removing duplicates, 5,018 articles remained. Upon reviewing titles and abstracts, 4,986 articles were excluded for not meeting the study theme. Full-text reviews and application of inclusion/exclusion criteria further excluded 8 articles for mismatched study subjects and 4 for inappropriate interventions. Ultimately, 20 articles (10–29) were included. The literature screening process is illustrated in Figure 1.

### 3.2 Characteristics of included studies

The basic characteristics of the included literature are shown in Table 1. The included studies covered 9 countries with a total of 5,248 patients and involved 4 types of interventions. Thirteen of the included studies were two-armed trials (10, 12, 15–22, 24, 27, 28) and 7 were three-armed trials (11, 13, 14, 23, 25, 26, 29).

**Table 1:** The general information of the included studies.

Author publication year	Country	Sample size (*n*)	Age (years)	Baseline data a: Weight b1: SBP b2: DBP c: Waist Circumference	Intervention Group 1	Intervention Group 2	Control group	Intervention duration
Sakane et al. (10)	Japan	39 vs. 39	52.5 ± 6.6 vs. 51.4 ± 6.4	a:-–	Use of KENPO-app, providing online face-to-face health consultation, setting personalized behavior goals for users, and reminding users to conduct daily self-monitoring.		Usual support	6 months
				b1:136.2 ± 17.5 vs. 138.3 ± 12.7				
				b2:–				
				c:–				
Appel et al. (11)	USA	268 vs. 269 vs. 273	50.2 ± 8.6 vs. 50.2 ± 9.3 vs. 49.5 ± 8.8	a:95.8 ± 17.0 vs. 96.2 ± 17.8 vs. 98.8 ± 19.3	At least 180 min of moderate-intensity physical activity per week.	1. DCSH diet	Usual care	18 months
				b1:134.2 ± 10.1 vs. 135.5 ± 9.2 vs. 134.9 ± 9.4		2. At least 180 min of moderate-intensity physical activity per week.		
				b2:84.8 ± 4.3 vs. 85.0 ± 4.1 vs. 84.6 ± 4.0				
				c:–				
Lisón et al. (12)	Spain	55 vs. 50	54.9 ± 8.3 vs. 51.4 ± 9.3	a:85.4 ± 12.0 vs. 81.1 ± 11.8	1. Exercise		Multimedia online intervention program	3 months
				b1:132.2 ± 14.2 vs. 128.5 ± 13.5	2. Low-calorie diet			
				b2:78.7 ± 8.1 vs. 75.8 ± 9.1	3. Multimedia online intervention			
				c:–				
Steffen et al. (13)	USA	35 vs. 42 vs. 15	47.5 ± 8.5 vs. 41.9 ± 10.3 vs. 46.1 ± 7.6	a:92.2 ± 17.6 vs. 91.5 ± 16.4 vs. 94.0 ± 14.8	1. Aerobic exercise 3–4 times per week	1. Aerobic exercise 3–4 times perweek	Usual care	6 months
				b1:143.2 ± 9.8 vs. 143.3 ± 12.3 vs. 138.6 ± 8.4		2. Dietary control		
				b2:93.7 ± 5.0 vs. 92.9 ± 5.2 vs. 93.4 ± 4.7	2. Normal diet	3. Weekly 30-min small group meetings (3–5 people)		
				c:–				
Blumenthal et al. (14)	USA	46 vs. 49 vs. 49	51.8 ± 10 vs. 52.3 ± 10 vs. 51.8 ± 9	a:92.6 ± 15 vs. 93.0 ± 14 vs. 93.9 ± 14	DASH diet alone	1. DASH diet	Usual care	2 weeks
				b1:138.0 ± 9.5 vs. 137.6 ± 9.0 vs. 138.7 ± 8.2		2. Exercise 3 times per week		
				b2:85.0 ± 5.8 vs. 86.1 ± 6.1 vs. 85.5 ± 6.8				
				c:–				
Nowson et al. (15)	USA	27 vs. 27	47.1 ± 10.3 vs. 48.8 ± 8.3	a:98.2 ± 1.9 vs. 88.2 ± 1.8				12 weeks
				b1:128.4 ± 2.0 vs. 130.9 ± 2.9	1. DASH diet		1. Low-fat diet advice	
				b2:84.0 ± 1.6 vs. 82.3 ± 1.6	2. Exercise advice		2. Exercise advice	
				c:–				
Ueki et al. (16)	Japan	13 vs. 22	56.9 ± 13.5 vs. 54.6 ± 11.7	a:76.4 ± 15.5 vs. 83.1 ± 21.0	Use of information and communication technology to upload data and receive online guidance from a dietitian.		Only received 3 face-to-face dietitian guidance sessions at the beginning.	12 weeks
				b1:148 ± 16 vs. 134 ± 11				
				b2:92 ± 12 vs. 84 ± 7				
				c:–				
Stuart et al. (17)	Australia	26 vs. 23	48.0 ± 5.88	a:98 ± 1 vs. 95 ± 1	1. Telephone support (1 introductory call + 1 follow-up call every 2 weeks, total 6 follow-up calls)		Usual care	12 weeks
				b1:126 ± 3 vs. 124 ± 3				
				b2:98 ± 1 vs. 95 ± 1				
				c:104.85 ± 1.3 vs. 106.36 ± 1.35	2. Exercise intervention (aerobic exercise + muscle strengthening training)			
Poulsen et al. (18)	Denmark	26 vs. 23	48.0 ± 5.9 vs. 48.0 ± 5.9	a:90.9 ± 19.3 vs. 91.8 ± 16.3	Adopted NDD diet		Usual diet	12 weeks
				b1:128.2 ± 15.0 vs. 128.6 ± 18.0				
				b2:77 ± 13.7 vs. 81 ± 9.5				
				c:101.1 ± 14.3 vs. 101.4 ± 11.8				
Fanaroff et al. (19)	USA	91 vs. 56	44.2 ± 13.0 vs. 40.8 ± 13.2	a:108.4 ± 21.3 vs. 106.6 ± 17.8	1. Daily two-way SMS reminders		1:16:8 time-restricted eating advice	26 weeks
				b1:141 ± 19.6 vs. 146 ± 15.0				
				b2:77 ± 13.7 vs. 81 ± 9.5	2. 16:8 time-restricted eating		SMS reminders	
				c:–	3. Buddy support			
Bennett et al. (20)	USA	20 vs. 17	60.0 ± 11.8 vs. 60.1 ± 10.3	a:99.7 ± 13.8 vs. 98.9 ± 14.4	1. Exercise for intervention group		Usual care	12 weeks
				b1:130.0 ± 17.6 vs. 130.1 ± 17.4	2. Diet			
				b2:81.9 ± 11.8 vs. 82.1 ± 11.6				
				c:115.0 ± 10.2 vs. 114.4 ± 10.2	3. Remote guidance			
Burke et al. (26)	Australia	176 vs. 175	50.9 ± 9.1 vs. 50.5 ± 8.7	a:84.2 ± 12 vs. 86.7 ± 13.3	1. DASH diet for intervention group		Usual care	12 months
				b1:125 ± 10 vs. 126 ± 11	2. At least 30 min of exercise daily			
				b2:76 ± 8 vs. 77 ± 7				
				c:93.7 ± 9.8 vs. 96.6 ± 10				
ter Bogt et al. (22)	Netherlands	123 vs. 118	57.1 ± 7.2 vs. 55.3 ± 7.5	a:–	Standardized computer-guided lifestyle counseling provided by nurse practitioners, including exercise advice, etc.		Usual care	12 months
				b1:145 ± 15.5 vs. 146 ± 18.5				
				b2:86 ± 8.2 vs. 87 ± 9.6				
				c:–				
Baer et al. (23)	USA	225 vs. 232	55.3 ± 7.7 vs. 56.9 ± 7.8	a:91.8 ± 14.4 vs. 91.6 ± 14.4 vs. 92.9 ± 13.8	Use of online platform	1. Use of online platform	Usual care	12 months
				b1:–		2. Support personnel		
				b2:–				
				c:–				
Bennett et al. (24)	USA	216 vs. 298 vs. 326	59.1 ± 8.8 vs. 60.1 ± 8.3 vs. 58.7 ± 8.6	a:100.60 ± 18.67 vs. 99.70 ± 16.29	Use of electronic health components to promote weight loss and hypertension self-management.		Usual care	12 months
				b1:128.50 ± 19.73 vs. 130.20 ± 18.89				
				b2:77.45 ± 13.77 vs. 79.34 ± 12.73				
				c:100.7 ± 11.5 vs. 100.5 ± 9.5				
Little et al. (25)	UK	180 vs. 185	54.6 ± 10.8 vs. 54.7 ± 11.0	a:104.38 vs. 102.4 vs. 102.93	1. Provision of 24 web-based series courses, with email reminders.	Provision of web courses and remote consultation	Evidence-based dietary advice and 6-month nurse follow-up	24 months
				b1:136.2 ± 17.5 vs. 138.3 ± 12.7	2. Three scheduled (and four optional) face-to-face nurse support meetings.			
				b2:–				
				c:-				
Hinderliter et al. (21)	USA	279 vs. 270 vs. 269	52.7 ± 13.3 vs. 54.7 ± 12.9 vs. 53.7 ± 13.2	a:92.6 ± 15 vs. 93.0 ± 14 vs. 93.9 ± 14	DASH diet alone	1. DASH diet	Usual care	12 months
				b1:138 ± 9 vs. 138 ± 9 vs. 139 ± 8		2. Supervised aerobic exercise 3 times per week		
				b2:86 ± 6 vs. 86 ± 6 vs. 85 ± 7				
				c:–				
Burke et al. (27)	Australia	46 vs. 49 vs. 49	51.8 ± 10 vs. 52.3 ± 10 vs. 51.8 ± 9	a:84.2 ± 10.8 vs. 86.7 ± 12.4	1. DASH diet for intervention group		Usual care	16 weeks
				b1:125 ± 10 vs. 128 ± 11				
				b2:76 ± 8 vs. 77 ± 7				
				c:93.8 ± 8.4 vs. 96.6 ± 9.3	2. Exercise advice			
Wright et al. (28)	New Zealand	123 vs. 118	57.1 ± 7.2 vs. 55.3 ± 7.5	a: 96.9 ± 7.4 vs. 94.8 ± 6.4	1. Low-fat plant-based diet		Usual care	4 months
				b1:132 ± 7 vs. 133 ± 6	2. Twice-weekly small group meetings			
				b2:78 ± 3 vs. 81 ± 3				
				c:110 ± 5 vs. 108 ± 4				
Wang YB et al. (29)	China	22 vs. 22	61.7 ± 4 vs. 59.38 ± 4.51	a:63.42 ± 6.03 vs. 66.65 ± 4.57	Supervised exercise 4 times per week		Usual care	12 weeks
				b1:145.47 ± 6.93 vs. 146.70 ± 6.34				
				b2:90.12 ± 7.32 vs. 90.65 ± 4.77				
				c:–				

Data are presented as mean ± standard deviation or as specified. SBP, Systolic Blood Pressure; DBP, Diastolic Blood Pressure. “–” indicates data not reported.

### 3.3 Quality assessment of included studies

**Figure 2:**
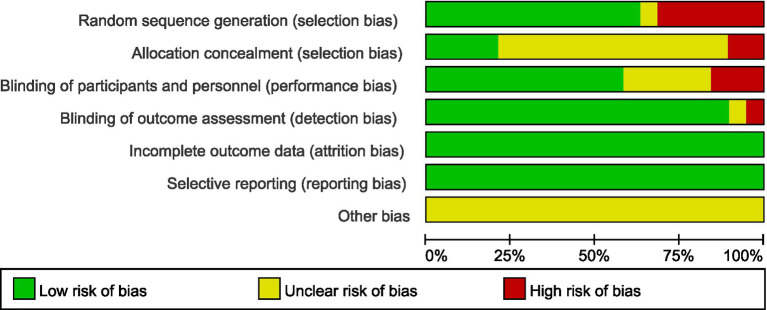
Risk of bias graph.

**Figure 3:**
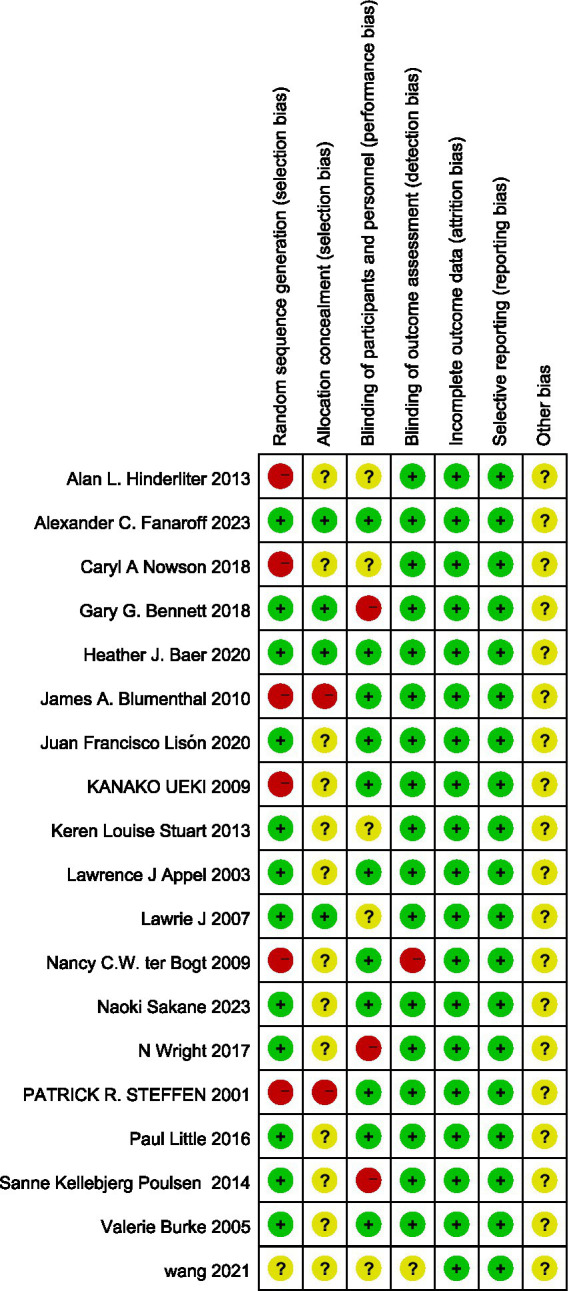
Risk of bias summary.

Seventeen of the included studies referred to a specific method of randomization of grouping, of which 1 (10) used random number table method and 9 (6, 11, 12, 14, 15, 17–28) used computer randomly generated grouping. 8 studies mentioned allocation concealment method, 1 (15) controlled random allocation scheme by telephone, 2 (17, 26) studies used same appearance envelopes, 5 (10, 14, 19, 25, 28) studies used web controlled random allocation scheme. 4 (12, 17, 25, 26) studies implemented double blinding, 4 (10, 11, 14, 28) studies implemented single blinding, and the rest did not specify whether blinding was implemented. The included studies ensured completeness of the outcome data and there was no selective reporting of data. 3 (17, 25, 26) studies had a quality grade of A, and the remaining studies had a grade of B. Literature quality assessment is shown in Figures 2, 3.

### 3.4 Baseline data

**Figure 4:**
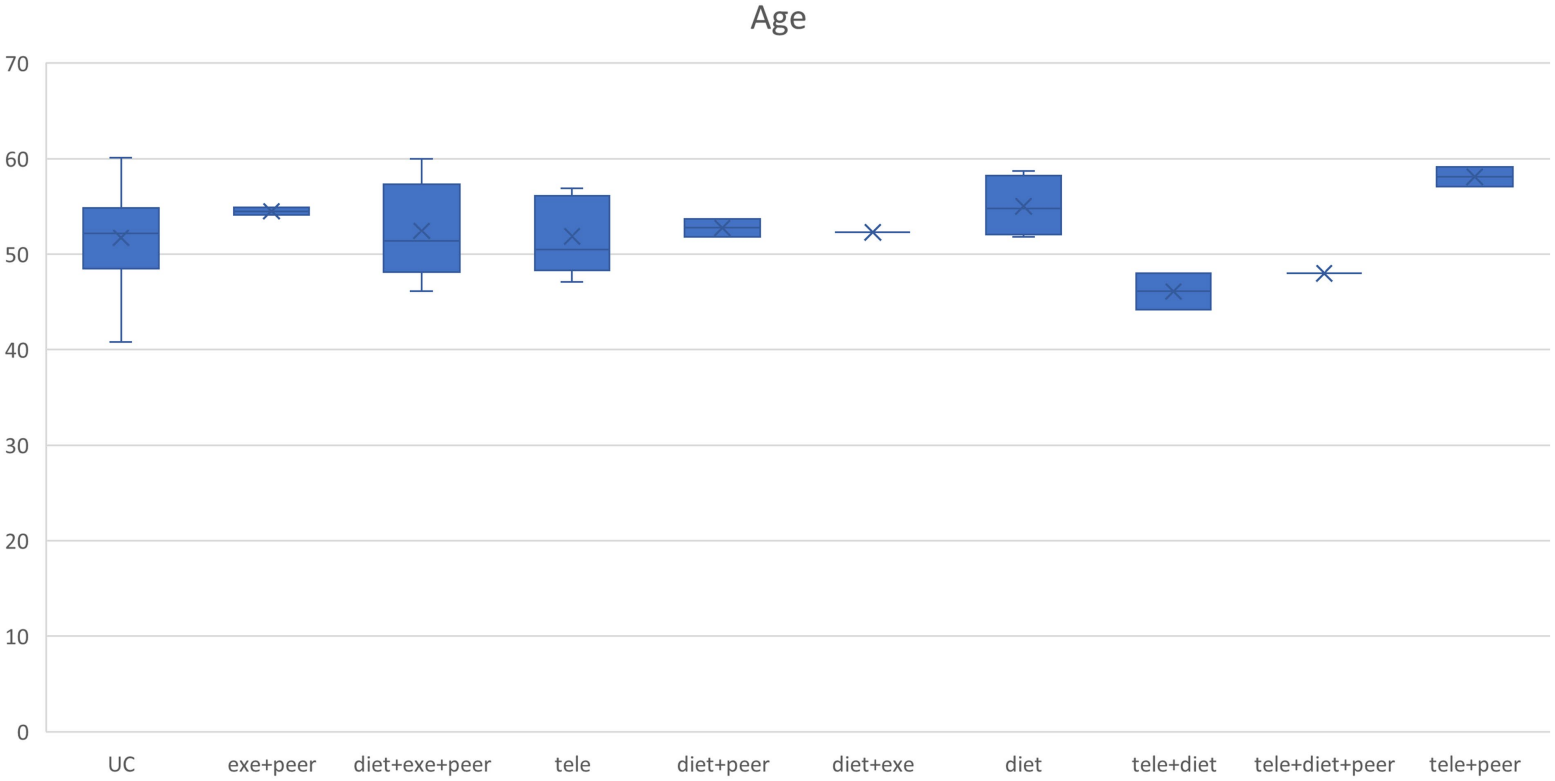
Age box chart.

**Figure 5:**
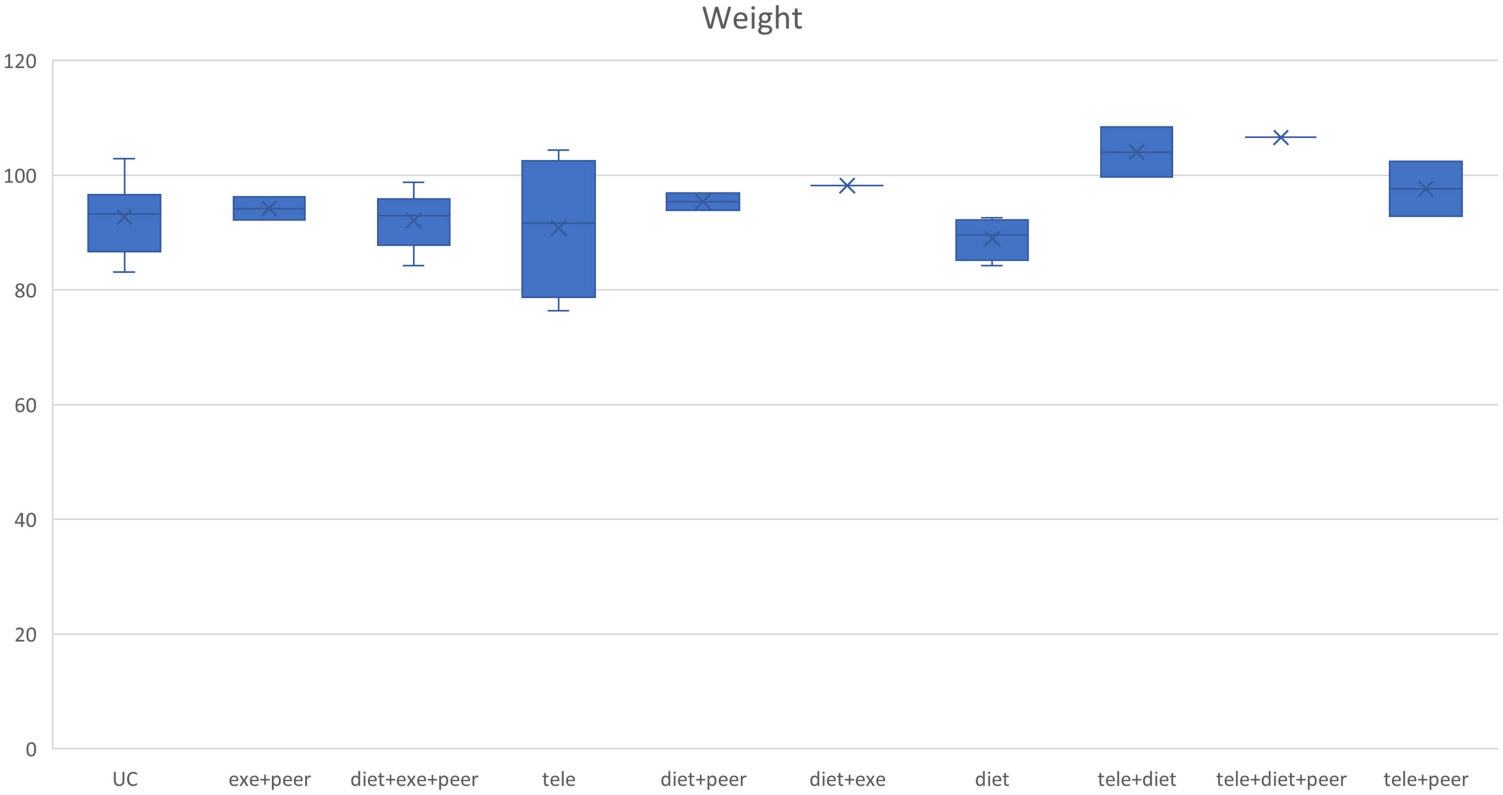
Weight box chart.

**Figure 6:**
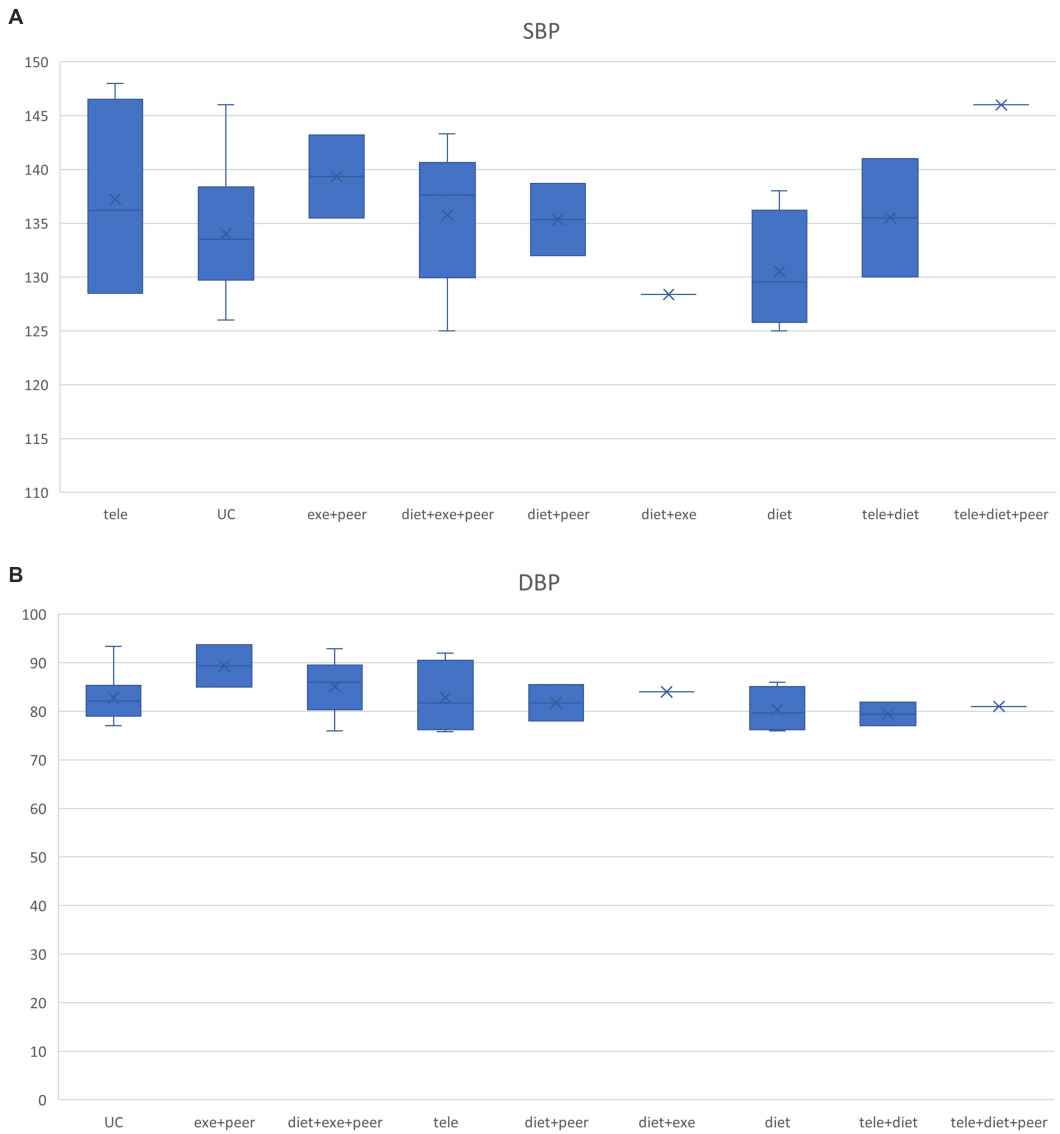
Blood pressure box chart. **(A)** SBP box chart, **(B)** DBP box chart.

This study extracted baseline characteristics (including age, blood pressure, and weight) from each group included in the research. Box plots depicting the distribution of baseline characteristics across multiple groups were generated according to different intervention protocols (see Figures 4–6). The box plot results indicate significant overlap between the boxes and whiskers of each group, demonstrating substantial similarity among most study populations. This satisfies the transitivity assumption required for indirect comparisons.

## 4 Outcome

### 4.1 Weight loss values

**Figure 7:**
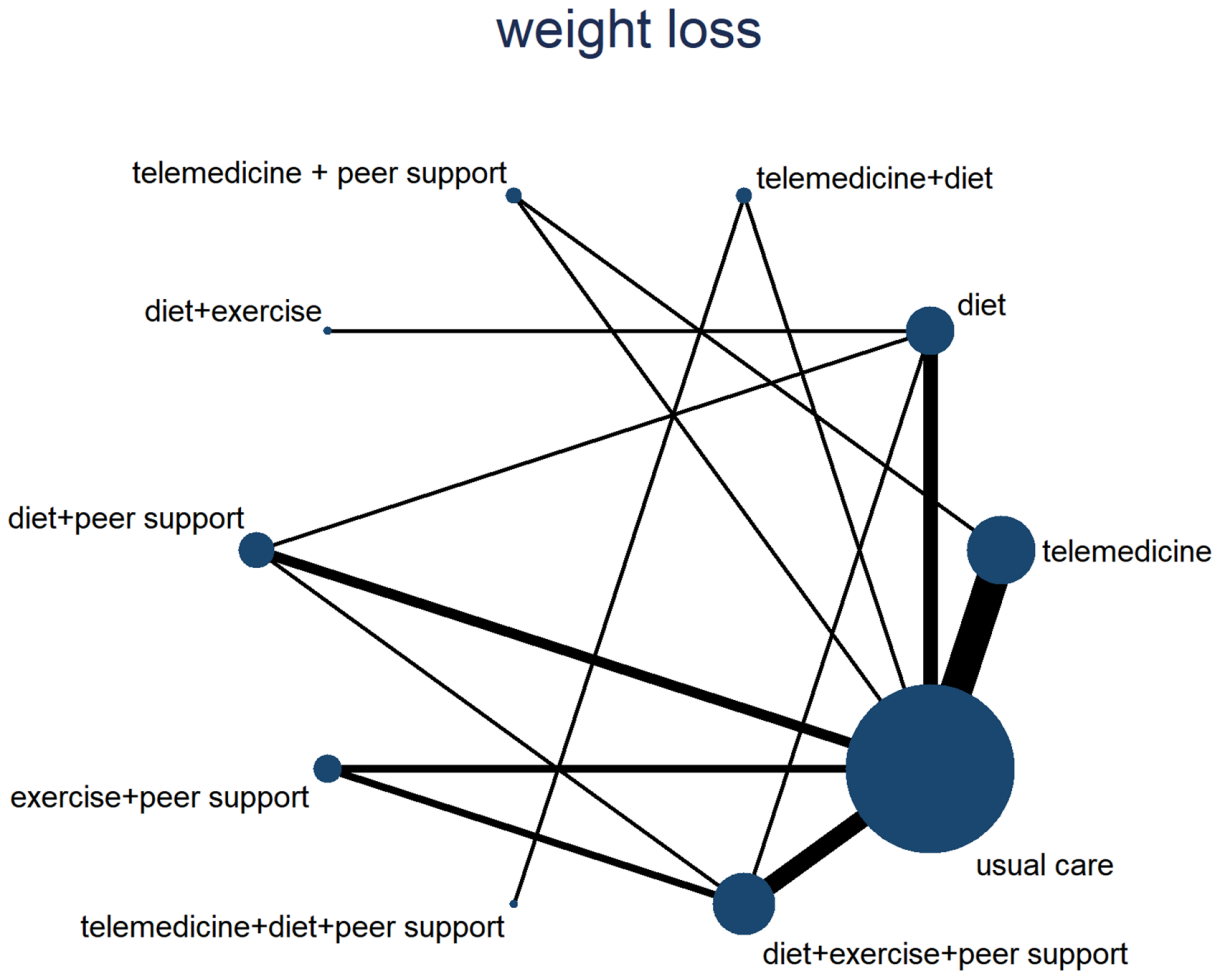
A network diagram for weight loss values.

**Figure 8:**
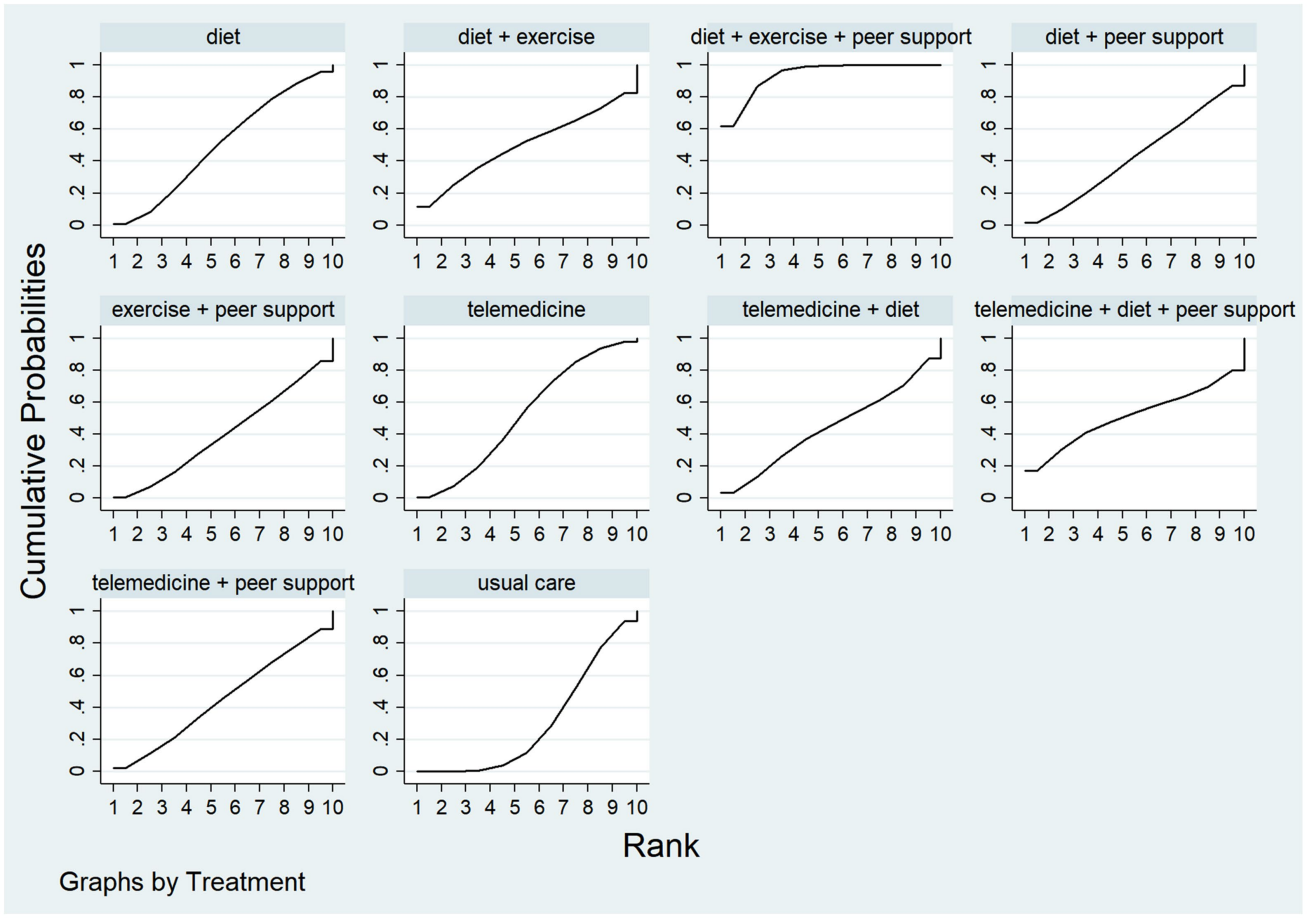
The probability ranking for weight loss values.

This study compared weight loss values across different interventions, incorporating 18 published studies. It compared 10 distinct intervention types and constructed a network diagram (see Figure 7). Following a global inconsistency test, *p* = 0.57 > 0.05. Local inconsistency was assessed using node-by-node analysis, showed no significant heterogeneity at any comparison node (*p* > 0.05), indicating consistency between direct and indirect comparisons. Loop inconsistency testing revealed no inconsistency within closed loops (p > 0.05). Therefore, the consistency model was adopted for effect size pooling. The probability analysis in Figure 8 suggests that diet + exercise + peer support may be the most effective weight loss intervention, with a surface curve of 61.9%. Table 2 presents the results of direct and interactive analyses for the 10 treatment measures, with bold font indicating significant differences.

**Table 2:** The network meta-analysis for weight loss values.

Diet + exercise + peer support									
2.95 (0.30, 5.59)	Telemedicine								
2.90 (−3.87, 9.68)	−0.04 (−6.74, 6.65)	Telemedicine + diet + peer support							
2.99 (0.02, 5.96)	0.04 (−3.02, 3.11)	0.09 (−6.86, 7.03)	Diet						
2.89 (−2.57, 8.35)	−0.05 (−5.56, 5.46)	−0.01 (−8.33, 8.31)	−0.09 (−4.68, 4.49)	Diet + exercise					
3.22 (−0.32, 6.76)	0.27 (−2.65, 3.19)	0.32 (−6.78, 7.42)	0.23 (−3.63, 4.10)	0.33 (−5.67, 6.32)	Telemedicine + peer support				
3.31 (−1.66, 8.29)	0.37 (−4.50, 5.24)	0.41 (−4.19, 5.01)	0.32 (−4.89, 5.53)	0.42 (−6.52, 7.36)	0.09 (−5.32, 5.50)	Telemedicine + diet			
3.30 (−0.03, 6.63)	0.35 (−3.15, 3.86)	0.40 (−6.75, 7.55)	0.31 (−3.60, 4.22)	0.41 (−5.61, 6.43)	0.08 (−4.14, 4.31)	−0.01 (−5.49, 5.47)	Diet + peer support		
3.52 (0.52, 6.51)	0.57 (−2.87, 4.00)	0.61 (−6.50, 7.73)	0.53 (−3.28, 4.33)	0.62 (−5.33, 6.58)	0.30 (−3.87, 4.46)	0.20 (−5.23, 5.64)	0.21 (−3.92, 4.35)	Exercise + peer support	
3.84 (1.83, 5.84)	0.89 (−0.84, 2.62)	0.93 (−5.53, 7.40)	0.85 (−1.68, 3.38)	0.94 (−4.29, 6.18)	0.62 (−2.30, 3.54)	0.52 (−4.03, 5.08)	0.54 (−2.52, 3.59)	0.32 (−2.65, 3.29)	Usual care

### 4.2 Reduction in blood pressure

**Figure 9:**
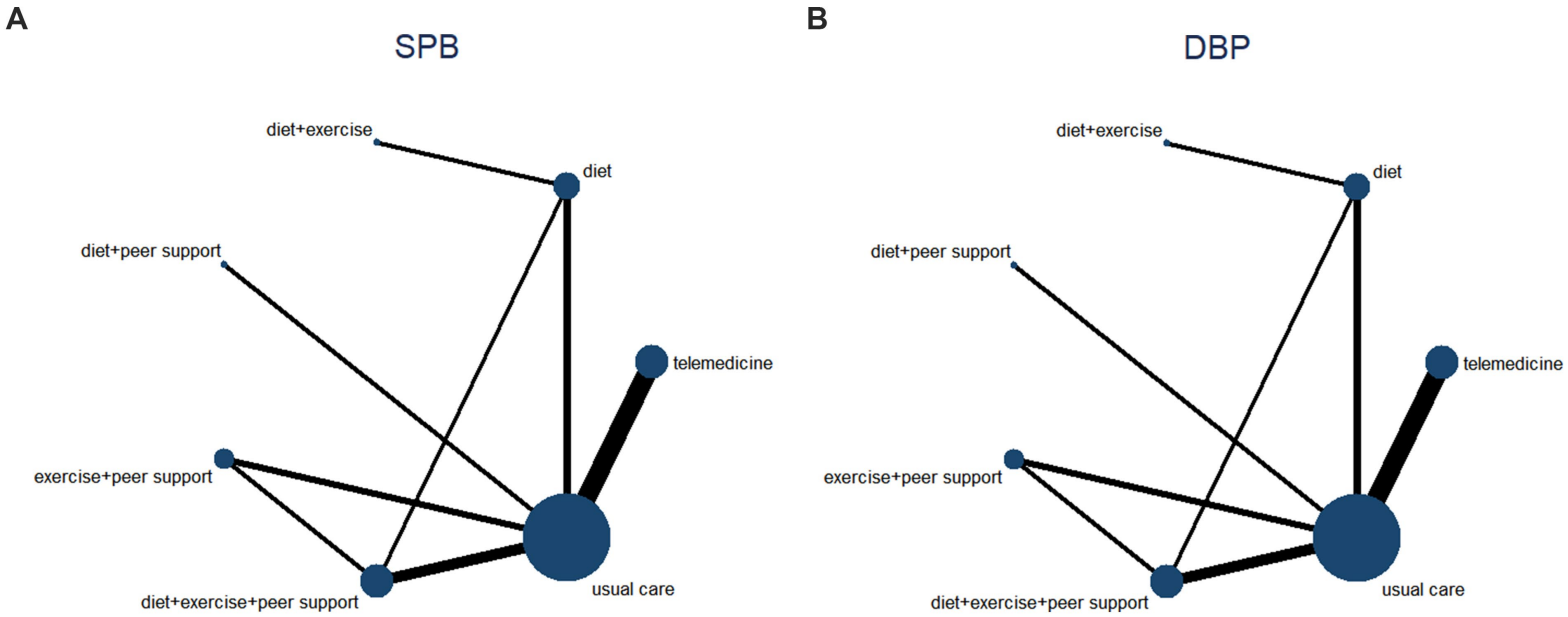
A network diagram for Reduction in BP. **(A)** A network diagram for Reduction in SBP, **(B)** A network diagram for Reduction in DBP.

**Figure 10:**
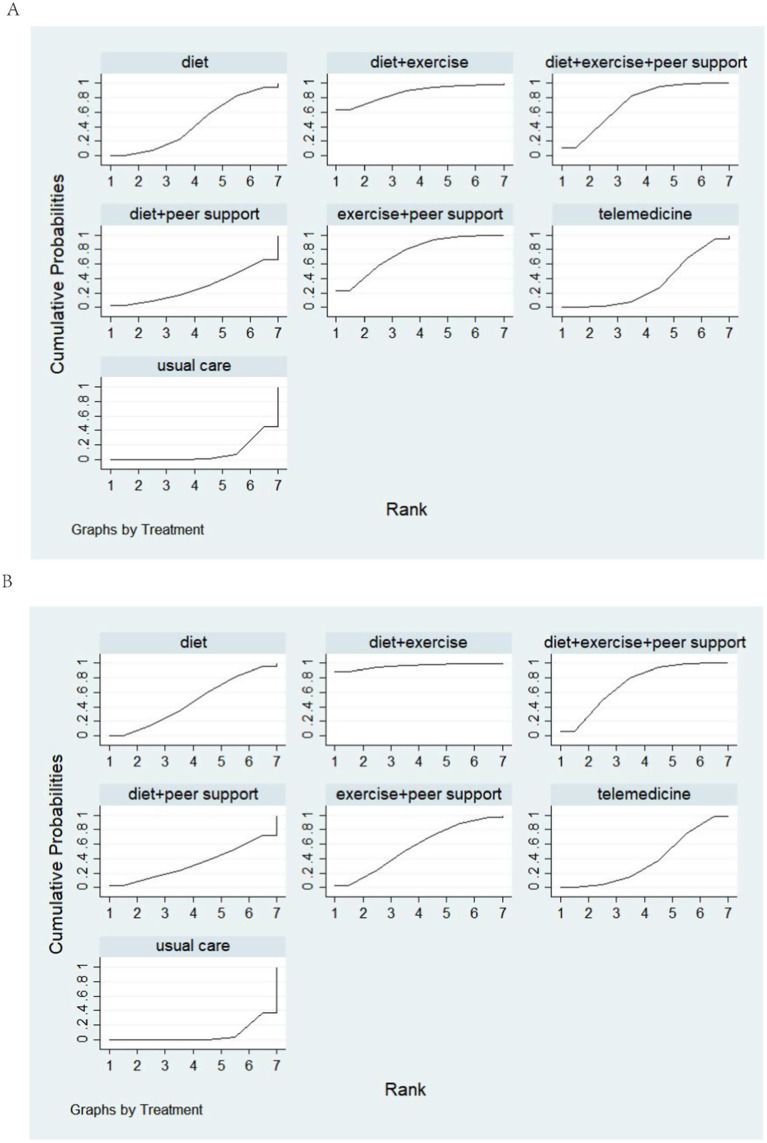
**(A)** The probability ranking for Reduction in SBP. **(B)** The probability ranking for Reduction in DBP.

This study compared the magnitude of systolic blood pressure reduction across different interventions. Twelve studies were included, comparing seven distinct intervention protocols. A network diagram is presented in Figure 9A. Following a global inconsistency test, *p* = 0.6 > 0.05. Local inconsistency was assessed using node-cutting analysis. Showed no significant heterogeneity at any comparison node (*p* > 0.05), indicating consistency between direct and indirect comparisons. Loop inconsistency testing revealed no inconsistency within closed loops (p > 0.05). Therefore, a consistency model was used for effect size pooling. Probability analysis in Figure 10A indicates that exercise plus dietary support demonstrated the most pronounced effect in reducing systolic blood pressure, with a 63.4% probability of being the optimal intervention. Table 3 presents the results of direct and interactive analyses for the seven treatment measures, with bold text denoting statistically significant differences.

**Table 3:** The network meta-analysis for reduction in systolic blood pressure.

Diet + exercise						
0.34 (−0.96, 1.64)	Exercise + peer support					
0.40 (−0.80, 1.60)	0.06 (−0.62, 0.74)	Diet + exercise + peer support				
0.77 (−0.21, 1.75)	0.43 (−0.42, 1.28)	0.37 (−0.32, 1.06)	Diet			
0.93 (−0.30, 2.15)	0.59 (−0.16, 1.33)	0.53 (−0.10, 1.15)	0.16 (−0.57, 0.89)	Telemedicine		
1.03 (−0.46, 2.53)	0.69 (−0.44, 1.83)	0.63 (−0.43, 1.70)	0.26 (−0.86, 1.39)	0.11 (−0.92, 1.13)	Diet + peer support	
1.22 (0.06, 2.37)	0.88 (0.25, 1.50)	0.82 (0.33, 1.30)	0.45 (−0.17, 1.06)	0.29 (−0.11, 0.69)	0.18 (−0.76, 1.13)	Usual care

This study compared the reduction in diastolic blood pressure across different interventions. Twelve studies were included, comparing seven distinct intervention protocols. A network diagram is presented in Figure 9B. Following a global inconsistency test, *p* = 0.6 > 0.05. Local inconsistency was assessed using node-cutting analysis. Showed no significant heterogeneity at any comparison node (*p* > 0.05), indicating consistency between direct and indirect comparisons. Loop inconsistency testing revealed no inconsistency within closed loops (p > 0.05). Therefore, the consistency model was adopted for effect size pooling. The probability analysis in Figure 10B indicates that exercise plus dietary support also demonstrated greater efficacy in reducing diastolic blood pressure, with an 88.8% probability of being the optimal intervention. Table 4 presents the results of direct and interactive analyses for the seven treatment measures, with bold font denoting statistically significant differences.

**Table 4:** The network meta-analysis for reduction in diastolic blood pressure.

Diet + exercise						
0.88 (−0.21, 1.98)	0.15 (−0.40, 0.69)	Exercise + peer support				
0.97 (0.12, 1.82)	0.23 (−0.33, 0.80)	0.09 (−0.60, 0.78)	Diet			
1.07 (0.03, 2.11)	0.33 (−0.17, 0.84)	0.19 (−0.41, 0.78)	0.10 (−0.50, 0.70)	Telemedicine		
1.13 (−0.14, 2.40)	0.39 (−0.49, 1.28)	0.25 (−0.69, 1.19)	0.16 (−0.78, 1.11)	0.06 (−0.80, 0.92)	Diet + peer support	
1.38 (0.39, 2.36)	0.64 (0.25, 1.03)	0.49 (−0.01, 0.99)	0.40 (−0.10, 0.91)	0.31 (−0.02, 0.63)	0.24 (−0.55, 1.04)	Usual care

### 4.3 Blood pressure reduction effectiveness rate

**Figure 11:**
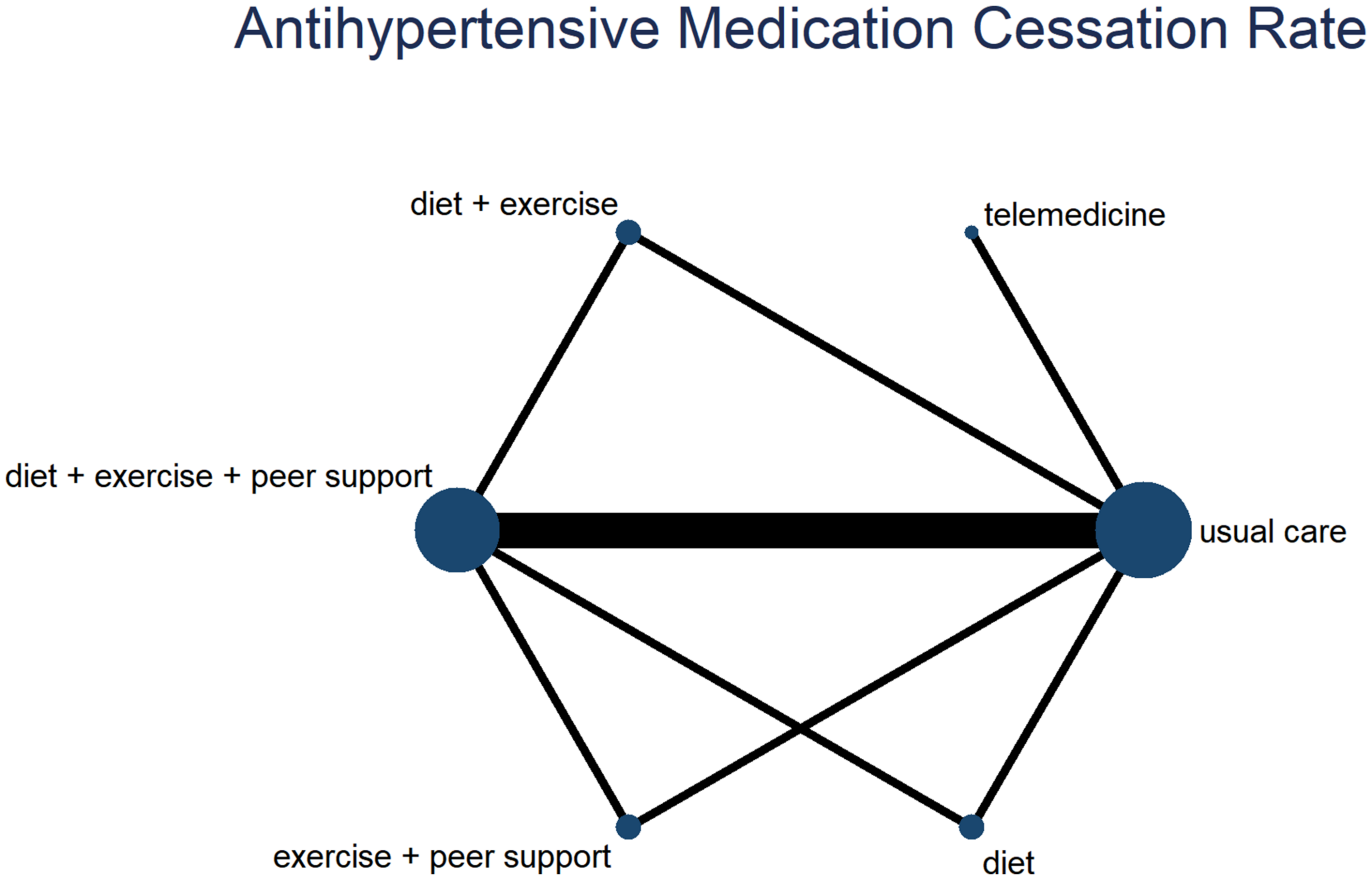
A network diagram of blood pressure reduction effectiveness rate.

**Figure 12:**
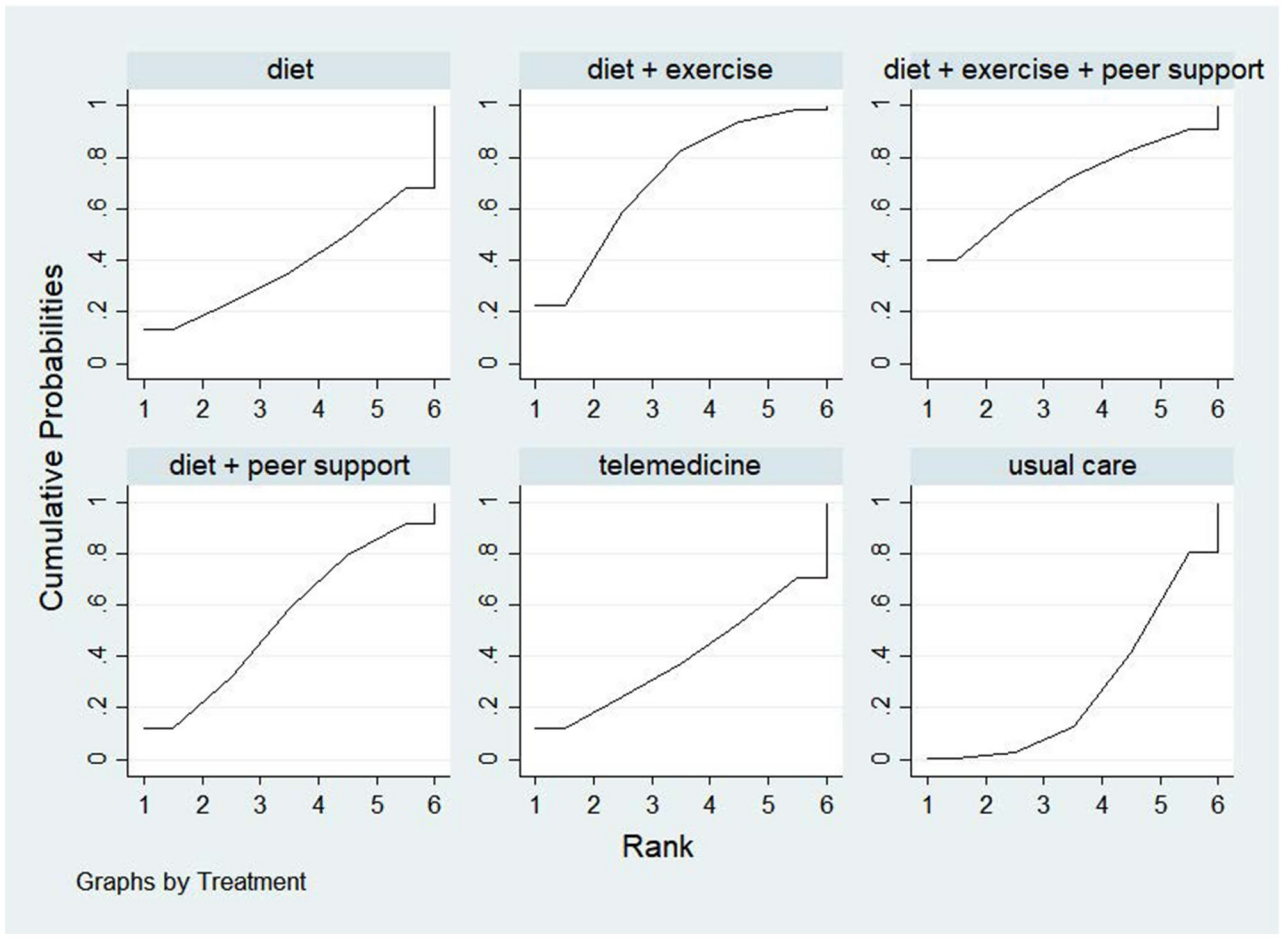
The probability ranking for blood pressure reduction effectiveness rate.

Five studies reported blood pressure reduction efficacy, comparing six distinct interventions and generating a network evidence diagram (Figure 11). Consistency testing via node splitting revealed no significant heterogeneity at any comparison node (*p* > 0.05), indicating consistency between direct and indirect comparisons. Thus, a consistency model was employed for effect size pooling. The probability analysis in Figure 12 indicates that exercise plus dietary intervention has a 36.9% probability of being the optimal strategy for helping overweight hypertensive patients achieve target blood pressure ranges, outperforming other interventions. Table 5 presents the direct and interactive analysis results for the six treatment measures, with bold text denoting statistically significant differences.

**Table 5:** The network meta-analysis for blood pressure reduction effectiveness rate.

Diet + exercise					
0.21 (−1.71, 2.14)	Diet + exercise + peer support				
0.42 (−1.06, 1.90)	0.20 (−2.07, 2.48)	Diet + peer support			
1.25 (−1.02, 3.52)	1.04 (−1.70, 3.77)	0.83 (−1.54, 3.21)	Diet		
1.27 (−0.96, 3.50)	1.06 (−1.64, 3.76)	0.85 (−1.49, 3.19)	0.02 (−2.61, 2.65)	Telemedicine	
1.55 (0.29, 2.81)	1.34 (−0.65, 3.32)	1.13 (−0.32, 2.58)	0.30 (−1.59, 2.18)	0.28 (−1.56, 2.12)	Usual care

### 4.4 Waist circumference reduction

**Figure 13:**
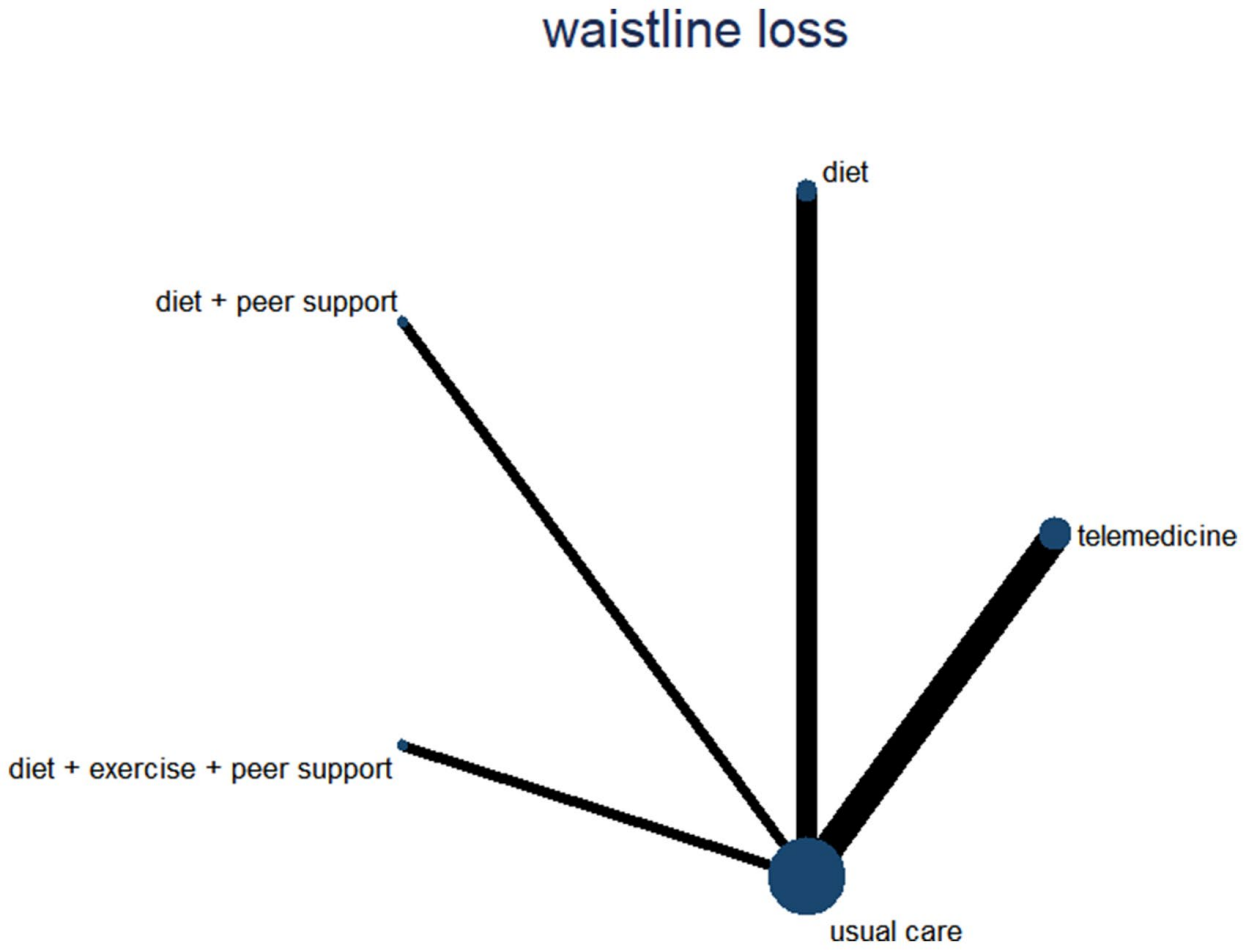
A network diagram for waist circumference reduction.

**Figure 14:**
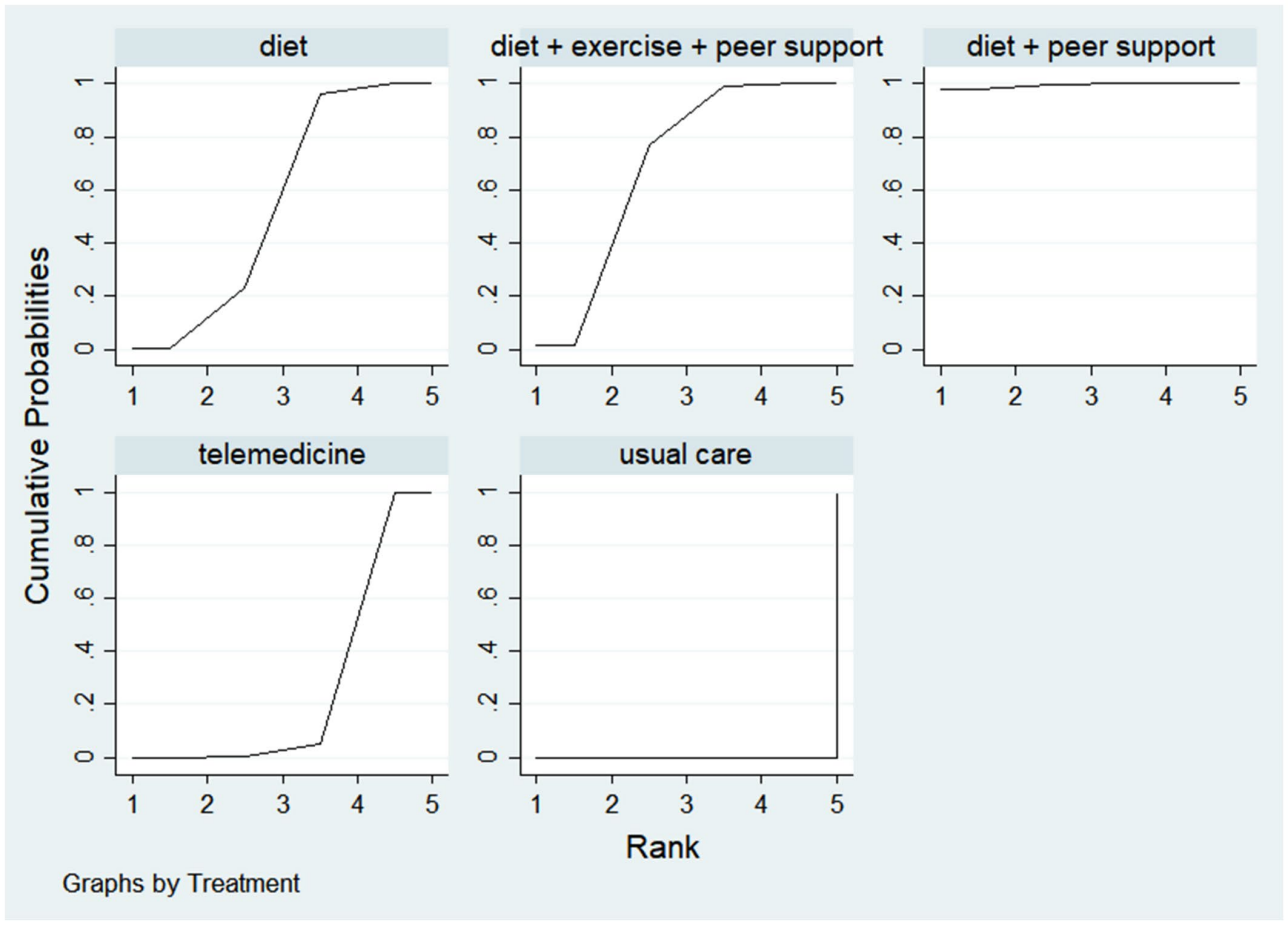
The probability ranking for waist circumference reduction.

Waist circumference, as a simple and effective indicator for assessing central obesity, has been proven to be a significant predictor of increased risk for hypertension, diabetes, dyslipidemia, metabolic syndrome, and coronary heart disease (30). This study included seven research articles comparing five different intervention measures and constructed a network evidence diagram (Figure 13). Since no closed loops appeared in the network evidence diagram, all comparisons between interventions were indirect comparisons, eliminating the need for consistency testing. The probability analysis in Figure 14 shows that the dietary intervention combined with peer support was superior to other interventions for reducing waist circumference, with a 98.1% probability of being the optimal approach. Table 6 presents the results of direct and mutual effect analyses for the six treatment measures, with bold text indicating statistically significant differences.

**Table 6:** The network meta-analysis for reduction in waist circumference reduction.

Diet + peer support				
0.66 (0.05, 1.26)	Diet + exercise + peer support			
0.79 (0.21, 1.37)	0.13 (−0.22, 0.49)	Diet		
1.01 (0.46, 1.57)	0.36 (0.04, 0.67)	0.22 (−0.03, 0.48)	Telemedicine	
1.26 (0.72, 1.79)	0.60 (0.32, 0.88)	0.47 (0.25, 0.68)	0.24 (0.10, 0.38)	Usual care

### 4.5 Publication bias

**Figure 15:**
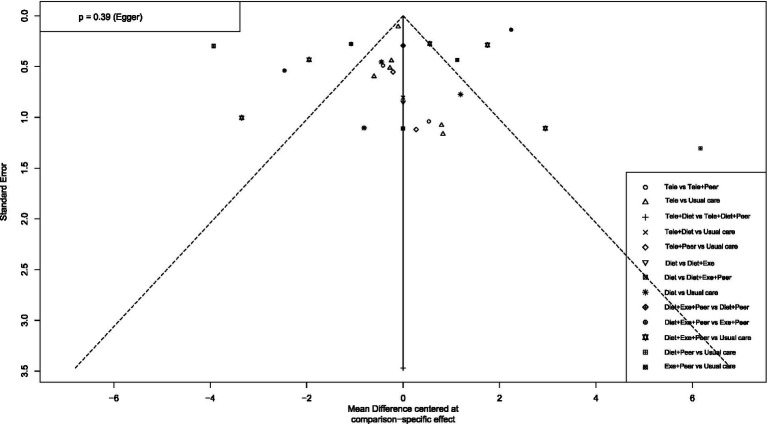
Funnel plot for weight loss.

**Figure 16:**
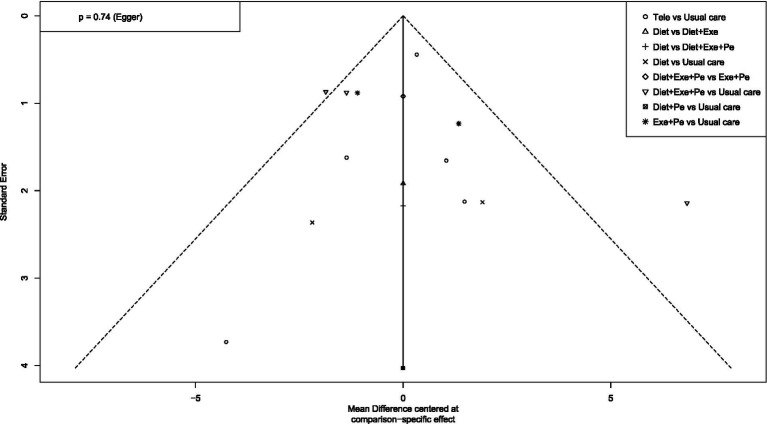
Funnel plot for SBP.

**Figure 17:**
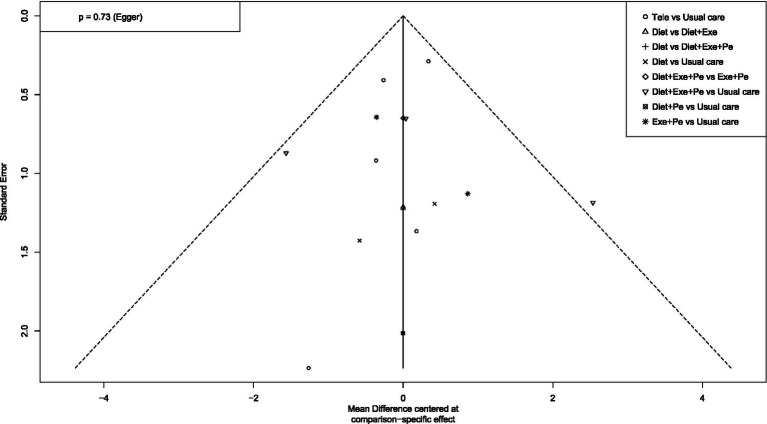
Funnel plot for DBP.

**Figure 18:**
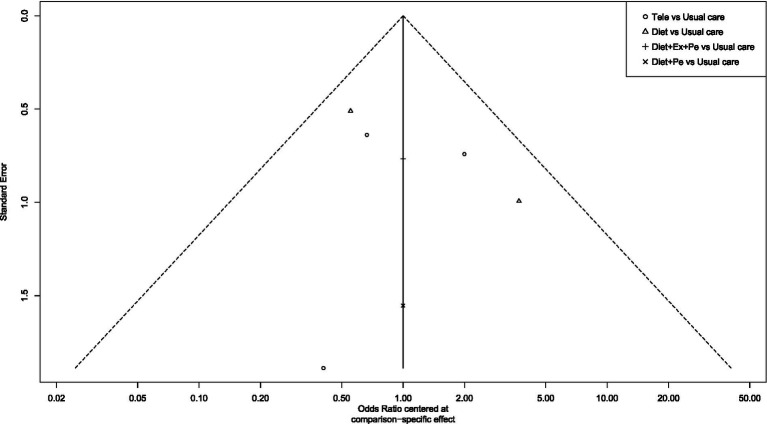
Funnel plot for blood pressure reduction effectiveness rate.

**Figure 19:**
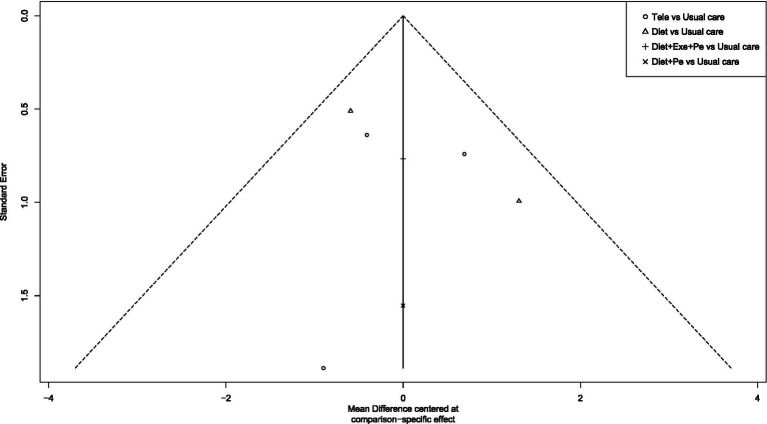
Funnel plot for waist circumference reduction.

To assess publication bias and the effect of small studies, this study first constructed funnel plots with comparison adjustments for primary outcome measures (Figures 15–19). The results showed that the funnel plots exhibited an overall symmetrical distribution, but some study nodes were scattered around the periphery of the funnel area, suggesting possible heterogeneity or the influence of individual small studies. For quantitative assessment, we further applied Egger’s regression test to outcome measures with sample sizes greater than 10. Results showed no statistically significant effects for weight reduction (*p* = 0.39), systolic blood pressure reduction (*p* = 0.74), or diastolic blood pressure reduction (*p* = 0.73) (*p* > 0.05). This indicates no significant publication bias within the network. However, the limited number of comparisons in this study may have constrained detection power. Furthermore, the distribution of some nodes in the funnel plot toward the periphery suggests that certain comparisons (e.g., diet + peer support vs. standard intervention; exercise + peer support vs. diet + exercise + peer support) may exhibit small-study effects or unmeasured heterogeneity. Although this asymmetry did not reach statistical significance in the overall Egger’s test, it could lead to overestimation of effect estimates for these specific intervention nodes, thereby influencing their relative positions in the SUCRA ranking. When interpreting the rankings, particular caution is warranted for interventions primarily relying on such evidence. In summary, although quantitative tests did not reveal significant publication bias, potential uncertainties from small-study effects or heterogeneity should be considered when interpreting results based on indirect evidence or comparisons involving these peripheral nodes (e.g., certain combined intervention regimens).

## 5 Discussion

This study confirms through a network meta-analysis that combined interventions or single interventions based on four lifestyle measures can produce certain improvements in both weight and blood pressure indicators for patients with obesity and hypertension. The development of obesity stems from a long-term imbalance between energy intake and expenditure, leading to excessive fat accumulation (31). Weight loss requires energy expenditure to exceed energy intake. Lifestyle modifications can increase energy expenditure and reduce intake through various means to achieve weight reduction. However, altering established habits remains challenging for most overweight individuals. A 14-year follow-up study revealed that under intensive lifestyle interventions, only 42 to 57% of participants across groups sustained long-term weight loss (32). Long-term weight management is influenced by multiple factors, and weight loss failure cannot be attributed solely to poor patient compliance. Meta-analyses indicate that consistent self-monitoring and high levels of exercise and weight loss self-efficacy are key predictors of sustained weight loss outcomes (33). Furthermore, intervention design characteristics (e.g., intervention type, duration), multidisciplinary team composition (e.g., inclusion of dietitians), and maintenance-phase follow-up intensity (e.g., ≥1 monthly professional consultation) significantly impact weight loss outcomes (34). Therefore, when developing lifestyle intervention strategies, the importance of long-term monitoring and sustained medical support should be fully considered to enhance the sustainability of intervention effects.

In terms of weight reduction, the combined intervention model of “diet + exercise + peer support” holds promise as a more effective management approach for overweight hypertensive patients. Dietary interventions (such as the DASH diet or time-restricted eating) focus on controlling energy intake, while exercise aims to increase energy expenditure, with both synergistically improving energy balance. However, for patients with long-standing unhealthy lifestyles, relying solely on dietary and exercise changes is often difficult to sustain over time, with effects diminishing gradually. Against this backdrop, peer support—an intervention strategy grounded in social cognitive theory—demonstrates unique value in promoting maintenance. Its mechanisms of action are primarily manifested in the following dimensions: First, by establishing a social support network that integrates emotional support, behavioral modeling, and practical assistance, it enhances patients’ self-efficacy and behavioral adherence (35). Second, it promotes the internalization of healthy behaviors and reduces psychological resistance through positive feedback and social recognition generated by group interactions (36). Third, particularly for populations with insufficient health literacy or socioeconomic resources, peer support compensates for structural support gaps, enhancing intervention accessibility (35). Clinical studies confirm (19) that peer support not only significantly improves physiological indicators like weight and blood pressure but also alleviates patients’ isolation in traditional interventions by establishing mutual aid networks, thereby strengthening the sustainability of long-term behavioral change. Compared to individualized guidance alone, peer support leverages social connections and group accountability mechanisms to reduce weight regain and provide dynamic, reality-based support for behavioral adjustments. This model deeply integrates bio-psycho-social medical principles, offering a comprehensive solution with clear evidence-based rationale and clinical feasibility for the long-term management of obesity and hypertension. However, it should be noted that this conclusion is partially based on indirect comparative evidence from network analyses. Furthermore, some studies supporting this node featured small sample sizes, high heterogeneity, and follow-up periods predominantly within 6 months. Therefore, while current data supports its short-term advantages, more robust evidence is required before it can be established as a universally applicable long-term management model.

Exercise combined with dietary intervention is a highly effective and prioritized approach for helping patients lower blood pressure and maintain it within target ranges. First, as a core non-pharmacological strategy for managing metabolic diseases, exercise intervention delivers clinical benefits primarily through two key dimensions: improving cardiovascular function and regulating body weight. Specifically, regarding cardiovascular protective mechanisms, regular aerobic exercise exerts antihypertensive effects through dual pathways: at the vascular structural level, it significantly reduces the arterial stiffness index (ASI) and decreases left atrial mass (37); at the vascular functional level, it enhances endothelium-dependent vasodilation by upregulating endothelial nitric oxide synthase (eNOS) expression (38). Concurrently, in weight management, exercise intervention synergizes with dietary control to produce metabolic effects: short-term, it enhances weight loss efficiency by increasing energy expenditure and fat oxidation rates (39); long-term, it reduces weight regain risk by improving energy metabolism homeostasis. Notably, epidemiological studies confirm a clear dose–response relationship between moderate-intensity aerobic exercise and weight loss outcomes (40). Therefore, from a clinical application perspective, while some research explores the potential value of resistance training, aerobic exercise (such as walking or jogging) has become the preferred exercise modality for weight management and cardiovascular risk prevention due to its superior safety, universality, and compliance advantages. This evidence-based exercise prescription strategy ensures scientific efficacy while maintaining feasibility in public health practice, providing crucial guidance for the comprehensive management of chronic metabolic diseases.

On the other hand, dietary control can improve vascular function by regulating the intake of various micronutrients, with the DASH diet pattern receiving the most robust evidence-based medical support. This pattern is characterized by achieving synergistic effects through multi-targeted intervention mechanisms: in blood pressure regulation, its high-potassium, high-fiber, and low-sodium dietary structure effectively improves vascular tone regulation; For weight management, fiber-rich foods reduce caloric intake by enhancing satiety and lowering energy density (41), while unsaturated fatty acids in nuts promote fat metabolism by activating *β*-oxidation pathways (42). Notably, low-fat dairy products may further optimize appetite regulation by modulating gastrointestinal hormones like CCK and GLP-1 (43), creating a cascade effect in metabolic control. Clinical studies confirm a dose–response relationship between the DASH diet’s blood pressure-lowering effects and weight changes (44), underscoring its central role in comprehensive hypertension management. Although emerging approaches like time-restricted eating show potential for short-term metabolic improvements, they face limitations due to risks of nutritional imbalance and compliance challenges. The DASH diet, with its scientific foundation, multisystem benefits, and sustainability advantages, remains the preferred nutritional intervention strategy recommended by international guidelines. In summary, this evidence-based practice not only provides a standardized approach for metabolic disease management but also establishes a theoretical foundation for optimizing personalized nutritional interventions. Therefore, combined dietary and exercise interventions demonstrate unique advantages in blood pressure reduction. Clinicians may consider incorporating such comprehensive interventions into non-pharmacological strategies for hypertension management in the future.

Digital health technologies, as an emerging intervention approach in chronic disease management, are gradually transforming traditional health management implementation pathways. Their core advantage lies in integrating health monitoring and behavioral interventions into patients’ daily lives through tools such as mobile applications and smart devices. This emerging model can be organically integrated with traditional lifestyle interventions like exercise and diet, forming complementary and synergistic management strategies that enhance the efficiency of comprehensive hypertension management. Particularly for patients with obesity and hypertension, digital health technologies demonstrate significant clinical value. Their core advantage lies in enabling real-time monitoring and data integration of physiological parameters through mobile health applications (e.g., KENPO-app) and wearable devices, providing evidence-based support for personalized interventions (11, 45). Existing evidence indicates that such telemedicine support systems can effectively improve patient weight and blood pressure control in the short term through dynamic feedback mechanisms and customized recommendations, while offering cost-effectiveness advantages (25). However, their long-term efficacy is constrained by fluctuations in patient adherence and heterogeneity in technological platforms (46), potentially stemming from three key factors: variations in patient digital literacy, insufficient algorithmic personalization, and the absence of sustained behavioral incentive mechanisms. To fully unlock the potential value of telemedicine, future development should prioritize: enhancing user-friendly human-machine interfaces, developing AI-based precision intervention algorithms, and establishing multidisciplinary support systems integrating clinical medicine, behavioral psychology, and information technology. These optimization strategies will help overcome current sustainability bottlenecks in remote health management, providing more effective digital solutions for chronic disease management.

In summary, this study provides quantitative evidence for comparing different lifestyle interventions through a network meta-analysis. However, certain limitations remain. First, the ranking relies partially on indirect comparative evidence, which carries higher uncertainty than direct comparisons. As indicated by the assessment of publication bias, the asymmetry within the network suggests that ranking results—particularly for intervention combinations involving peripheral nodes—may be sensitive to small-study effects or heterogeneity. Therefore, clinical decision-making should comprehensively consider effect sizes, confidence intervals, and the robustness of rankings, rather than relying solely on ranking order. Second, the interventions included in this study exhibit clinical heterogeneity in terms of intervention intensity (e.g., exercise frequency), delivery formats (e.g., peer support models), and baseline characteristics of the populations. Although the statistical model partially accounted for these differences, they may influence the transferability of effects. For instance, the ‘diet + exercise’ combination showing optimal results under high-intensity supervision may yield diminished effects in community settings. This suggests that SUCRA rankings reflect ‘average’ relative effects, necessitating adjustments based on local resources and patient characteristics during clinical implementation. Furthermore, this network lacks direct comparisons for some key contrasts (e.g., ‘diet + exercise + remote support’ vs. ‘diet + exercise + peer support’), making their rankings highly dependent on indirect evidence. The validity of indirect comparisons relies on the ‘consistency hypothesis,’ which may be undermined by clinical heterogeneity. Consequently, the certainty of conclusions for such rankings—primarily based on indirect evidence—is lower than for comparisons supported by direct evidence. Additionally, the vast majority of interventions in this study had follow-up periods shorter than 12 months, limiting our ability to assess long-term maintenance effects. Waning of behavioral intervention effects is a common phenomenon. Therefore, current rankings may reflect short- to medium-term effectiveness more accurately. For chronic conditions requiring long-term management, implementing top-ranked intervention strategies necessitates planning for reinforcement or maintenance phases.

## 6 Limitations

(I) The scope of literature retrieval is confined to Chinese and English databases, which may lead to the exclusion of some relevant studies, thereby posing a risk of incomplete retrieval; (II) The included literature spans multiple countries and regions, which may introduce bias due to variations in race, culture, and socioeconomic backgrounds; (III) Heterogeneity in intervention duration, baseline characteristics of the study populations, and intervention intensity among similar studies may impact the generalizability of the findings. (IV) Some studies incorporated a multi-arm trial design, where sharing the same control group may introduce potential confounding factors, leading to uncertainty in estimating the independent effects of different interventions. Future research should broaden the scope of literature retrieval, control for confounding variables, and validate the long-term efficacy and applicability of the aforementioned interventions through multi-center randomized controlled trials.

## 7 Conclusion

This systematic analysis of various lifestyle interventions suggests that dietary modifications, exercise, peer support, and telemedicine may all yield benefits in managing patients with hypertension and obesity. Among these, the combined intervention model integrating diet, exercise, and peer support shows a potential trend in promoting behavioral change and sustaining weight loss effects based on current evidence. However, it is important to note that current evidence has certain limitations. The findings of this analysis are influenced by factors such as heterogeneity among included studies, partial reliance on indirect evidence, and short follow-up periods in some studies, leaving long-term effects unclear. Among these, the combined intervention model integrating diet, exercise, and peer support appeared relatively more favorable in our analysis, suggesting a potential trend for promoting behavioral change. However, this observation is preliminary. Given the limitations noted above, including heterogeneity and the use of indirect evidence, definitive conclusions regarding the superiority of any single strategy cannot be drawn. Future high-quality, long-term RCTs with head-to-head comparisons are needed to confirm these findings and inform optimal clinical strategies.

## Data Availability

The original contributions presented in the study are included in the article/supplementary material, further inquiries can be directed to the corresponding authors.
